# Perspectives of healthcare workers on the integration of overdose detection technologies in acute care settings

**DOI:** 10.1186/s13722-023-00433-7

**Published:** 2024-01-12

**Authors:** William Rioux, Kyle Kilby, Stephanie Jones, Pamela Joshi, Stephanie Vandenberg, S. Monty Ghosh

**Affiliations:** 1 Department of Medicine, Faculty of Medicine & Dentistry, University of Alberta, Edmonton, AB Canada; 2 Department of Internal Medicine, Faculty of Medicine & Dentistry, University of Calgary, Calgary, AB Canada; 3 Three Hive Consulting, Vancouver, BC Canada; 4 Provincial Perinatal Substance Use Program, BC Women’s Hospital & Health Center, Provincial Health Services Authority, Vancouver, Canada; 5 Cumming School of Medicine, University of Calgary, Calgary, AB Canada; 6 Department of Internal Medicine, Faculty of Medicine & Dentistry, University of Alberta, Edmonton, AB Canada

**Keywords:** Opioid, Overdose, Acute care, Overdose detection technologies, Harm reduction

## Abstract

**Background:**

People who use drugs (PWUD) face disproportionately high rates of hospitalizations and patient-initiated discharge (leaving against medical advice), explained by a combination of stigma, withdrawal, judgment, blame, and improper pain management. In addition, evidence has shown that despite abstinence-based policies within healthcare settings, PWUD continue to use their substances in healthcare environments often hidden away from hospital staff, resulting in fatalities. Various novel overdose detection technologies (ODTs) have been developed with early adoption in a few settings to reduce the morbidity and mortality from risky substance use patterns within healthcare environments. Our study aimed to gain the perspectives of healthcare workers across Canada on implementing ODTs within these settings.

**Method:**

We used purposive and snowball sampling to recruit 16 healthcare professionals to participate in semi-structured interviews completed by two evaluators. Interview transcripts were analyzed using thematic analysis to identify key themes and subthemes.

**Results:**

Participants recognized ODTs as a potentially feasible solution for increasing the safety of PWUD in healthcare settings. Our results suggest the mixed ability of these services to decrease stigma and build rapport with PWUD. Participants further highlighted barriers to implementing these services, including pre-established policies, legal recourse, and coordination of emergency responses to suspected overdoses. Lastly, participants highlight that ODTs should only be one part of a multifaceted approach to reducing harm in healthcare settings and could currently be integrated into discharge planning.

**Conclusion:**

Healthcare professionals from across Canada found ODTs to be an acceptable intervention, but only as part of a larger suite of harm reduction interventions to reduce the harms associated with illicit drug use in healthcare settings. In contrast, participants noted institutional policies, stigma on behalf of healthcare workers and leadership would present significant challenges to their uptake and dissemination.

**Supplementary Information:**

The online version contains supplementary material available at 10.1186/s13722-023-00433-7.

## Introduction

North America is caught in the grips of an ongoing overdose epidemic, exacerbated by an increasingly toxic drug supply [1]. Alongside decades-high rates of substance-related mortality in Canada, hospitalization rates for overdose have seen a large increase since the declaration of the national public health emergency [2]. Indeed, people who use drugs (PWUD) interact with emergency departments and face hospitalization rates 4.8 and 7.1 times higher than the general population, respectively [3]. Within these interactions, the use of unregulated substances in the hospital is common. Between 34 and 44% of individuals hospitalized in the United States (U.S.) for injection-related infections report using illicit drugs during their hospital stay [4–6]. This statistic is echoed within the Canadian setting, wherein a cohort of 1028 PWUD in Vancouver who had experienced > 1 hospitalization, 43.9% of individuals reported using drugs during their hospital stay [7]. Despite the high prevalence of substance use in these settings, many hospitals enforce abstinence-based policies, which result in the adoption of risky behaviours (including using drugs alone, concealing use, and sharing and reusing supplies), increasing the risk of overdose and death [6–9]. Furthermore, people who inject drugs are significantly more likely to self-initiate discharge (leave hospitals against medical advice) compared to those who do not inject drugs [10, 11]. We chose to use the former term throughout as the authors believe that it is more patient-centered. This has partly been explained by improper pain management, continued cravings, withdrawal management, stigmatizing treatment, and shaming from healthcare providers [11, 12]. Moreover, overdoses/drug poisonings continue to occur within hospital settings, resulting in increased psychological and moral distress for staff who respond to overdose or recognize the presence of an overdose risk [13].

Currently, hospital-based supervised consumption sites (SCS) have been explored as a potential solution to this problem but remain limited in their availability. PWUD identified hospital-based SCS as a strategy they would endorse for promoting retention and patient-centred care [14] however, only 5 of these facilities exist globally [15]. This indicates a significant gap in care for many PWUD. Some jurisdictions have also explored episodic overdose prevention services as well as an alternative to SCS’ to support overdose prevention services offered in flexible settings based on need [16].

While the efficacy of these services continues to be evaluated, other solutions should be examined to mitigate the harms associated with illicit substance use in acute care settings, particularly in low-resource healthcare systems. A recent study in the United States recorded 357 opioid overdoses in the bathrooms of a single medical center over a three-year period (2016–2018), resulting in seven fatalities [17]. Similarly, over the course of a one-year period (2020–2021) in the United Kingdom, 42 patients died using substances during hospital admission, according to the coroner reports database [18]. Furthermore, individuals who are isolated for infection control in the hospital or are immobilized may not be able to transport themselves to a physical SCS, and thus warranting harm reduction support be brought to them. Lastly, not all patients disclose their substance use [19], and having resources available for those who do not may avoid gaps in care.

One proposed method to address this gap is the implementation of Overdose Detection Technologies (ODTs). These services include telephone lines upon which individuals can use their substances and operators can activate emergency services in the event of a drug poisoning. Examples of ODTs are Canada’s National Overdose Response Service (NORS) and the U.S.-based Never-Use Alone hotline [20, 21]. Other services include smartphone apps, buttons, and reverse motion sensors that can be used to alert appropriate individuals in the event that an individual becomes unresponsive or experiences an overdose. These technologies are summarized in various recent reviews [22–25] and show promise in being able to reduce the harms associated with solitary illicit drug use [26–28].

In efforts to evaluate how these services may be integrated into acute care settings, we have conducted a qualitative study to understand the perceptions of diverse healthcare providers crossing interdisciplinary and provincial boundaries in Canada. Our study aims to determine the perceptions of these stakeholders around the feasibility and acceptability of implementing these interventions within acute healthcare settings.

## Methods

Semi-structured telephone interviews were conducted with 16 healthcare professionals (including nurses, healthcare administration, and physicians), as described in Table 1. Individuals were identified using snowball and convenience sampling from participants across Canada and through existing networks of physicians who work in addiction medicine and currently work in acute care settings through the principal investigator (M.G.). These individuals were asked to participate in the study and asked to extend the invitation to their broader clinical and administrative team who work in addictions as well. Overall, 33 participants were identified in British Columbia, Alberta, Manitoba, and Ontario and invited to participate in the interviews, however, the invitations were forwarded to their other colleagues as well, broadening recruitment. Data was collected from February to March 2022 and from December 2022 to January 2023 to ensure thematic saturation was reached. Inclusion criteria required participants to be residents of Canada, 18 years of age or older, communicate effectively in English, provide informed verbal consent, and be currently practicing healthcare workers who work in healthcare settings. We selected interview participants who both work in acute care settings and who may have leadership and management roles whose buy-in would be required to enact these services in acute care settings. Before the interviews, all participants were provided with an information package. Interviews conducted over the telephone were approximately 20 to 60 min in length, conducted by two female evaluators with Masters level training from a third-party research consulting firm (SJ, LA). Except for 2 interviewees, there was no previously established relationship between evaluators and interview participants, and only the interviewer and interviewee were present on the call. No repeat interviews were conducted. Interviews were recorded using TapeACall and transcribed using a third-party transcription service. No field notes were taken for this analysis, and transcripts were not returned to participants for comment or correction.

**Table 1:** Participant characteristics

Participant Number	Province/Territory	Urban or Rural	Role
P1	AB	Urban	Registered Nurse Harm
P2	AB	Urban	Primary Care Physician
P3	AB	Urban	Nurse Educator
P4	BC	Urban	Clinical educator
P5	AB	Rural	Registered Nurse
P6	AB	Urban	Registered Nurse
P7	AB	Urban	Manager
P8	AB	Urban	Manager
P9	AB	Urban	Manager
P10	NS	Rural	Manager
P11	ON	Urban	Resident
P12	AB	Urban	Registered Nurse
P13	AB	Urban	Peer support worker
P14	BC	Both	Project lead
P15	BC	Rural	Regional Nursing Lead
P16	BC	Urban	Program Director

The study was conducted as part of a quality improvement project and received ethical approval from the University of Calgary Conjoint Health Research Ethics Board (REB21-1655). The COREQ framework was used to guide the reporting of results.

Interview guides were constructed by the research team in collaboration with ODT operators (From NORS/the Brave App), individuals with lived experience of substance use, and a qualitative research consulting firm (Three Hive) that conducted the interviews. As part of the interview guide all participants were provided with various examples of ODTs.

### The model for ODTs in acute care settings

**Fig. 1:**
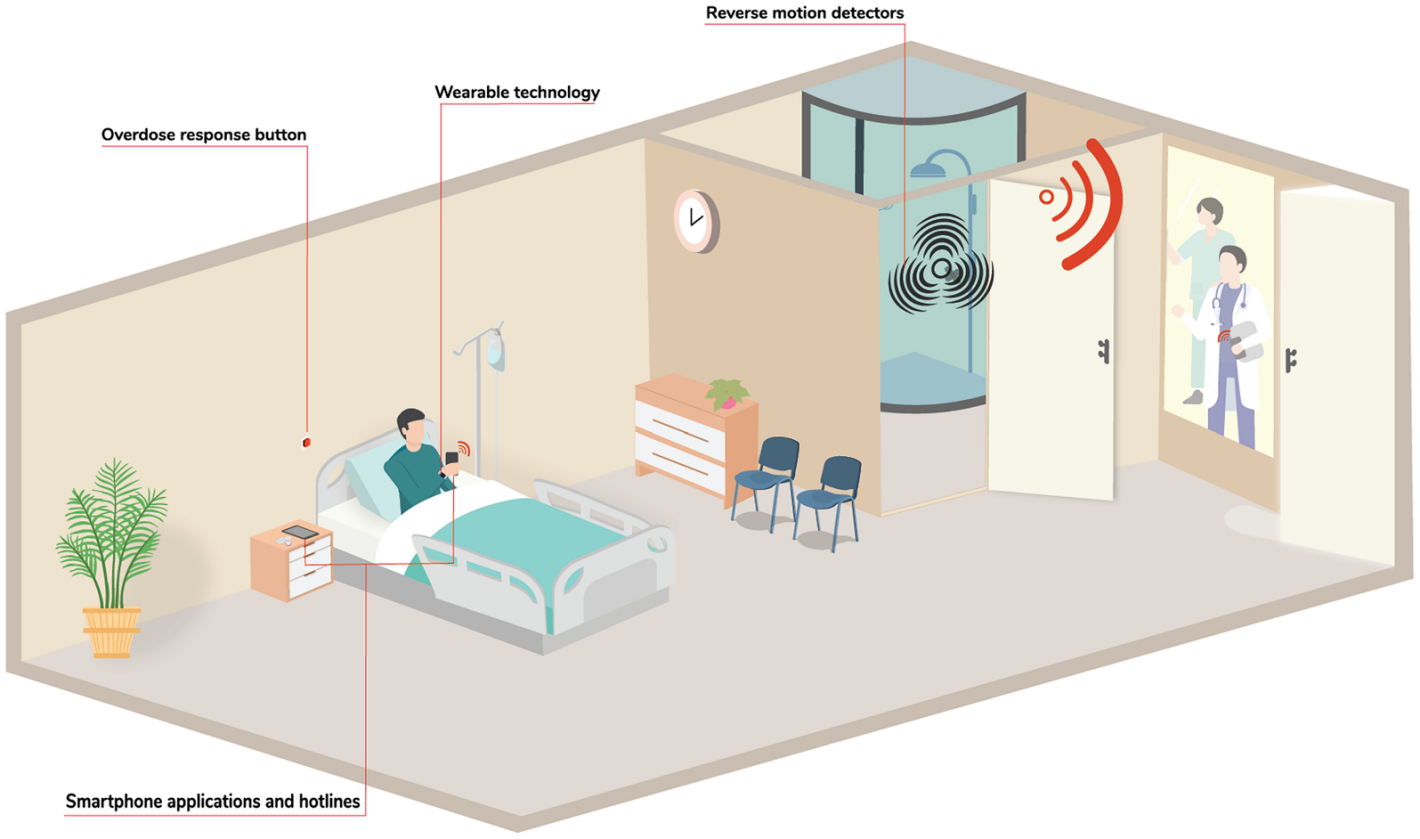
The Model for ODTs in acute care settings

Several different models of implementing technologies in acute care settings were presented. This included having phones or tablets available to patients in the privacy of their own rooms where they could contact smartphone-based ODTs. Brief concepts, such as buttons in hospitals, as well as reverse motion detectors in hospital rooms, were also presented for discussion. The buttons are operated by an individual pressing the button once to alert someone to check on them shortly or multiple times to indicate an acute emergency. Reverse motion detectors are activated once someone is in a room and sense if movement stops within it while the person remains inside. If movement stops, an alert is sounded or sent. While not included in the interview guide or discussion, we have also included wearable technologies that could or are currently used within acute care settings within Fig. 1.

Thematic analysis was used to inductively identify themes and subthemes. Qualitative results were encoded via thematic analysis, identifying common themes across participant responses. In the first three transcripts, coding was compared by the two evaluators to refine a codebook and ensure consistency, after which they coded the transcripts independently. To maintain coding congruence, each evaluator reviewed transcripts coded by their counterpart through *Dedoose.* Codes were developed using Proctor’s framework through a joint evaluator agreement and kept in a codebook, which was updated in real-time. Coding uncertainties were discussed between the two evaluators to ensure consensus. Once initial coding was complete, the evaluators reviewed a representative sample of coded quotations for each theme with a consulting project manager (KM, with Master’s level training in qualitative methods). Interviews were conducted until thematic saturation was reached. Triangulation of results was conducted by reviewing results with clinician contacts, including the PI (MG).

## Results

Overall, our results determine that while there are likely advantages to implementing ODTs within acute and primary care settings, there are major barriers to its implementation. Specifically, the feasibility of implementation and acceptance amongst patients and providers was questioned. Additionally, there are systems-based barriers that impact its implementation. Representative quotes are highlighted within the main text, and quotes supporting each subsection can be found in Additional file 1.

### *Section 1: Perspectives on patient care*

#### Subtheme 1: Acceptability, access, and patient safety around ODTs in acute care settings

When examining the utilization of these services in acute care settings, participants thought that this would help reduce the harm from substance use and prevent patient-initiated discharges. It was felt that by providing a supportive environment where patients had the option of using substances safely while in the hospital, patients were more likely to stay engaged with the acute care health care team for their medical needs, and less likely to self-discharge from the hospital secondary to their substance use. These issues include concerns when patients have cravings for substances or go through active withdrawals.

However, interviewees brought up the difficulties in phone and technology access for this population as a barrier to accessing these services, particularly the hotline and phone app services, especially if they were using their own phones. *“I would see it as a benefit to their safety and wellbeing. They’re going to use [substances] whether we want them to or not so how can we better facilitate their medical care if they are going to use… Anything that could promote them staying on the unit more I’m all behind…And if this service could do that well then by all means I think we should give it a go.” P13 (Urban peer support worker).*

*The barrier always will be phones for patients. I think a lot of people have pay-as-you-go phones and so don’t always have necessarily – they don’t have data or the ability to always have apps but then also they just don’t have phones too. P12 (Urban Registered Nurse).*

#### Subtheme 2: Rapport building with PWUD

While some technology pieces, including reverse motion detectors and buttons, directly alert on-site health care staff, other forms of technology, such as mobile overdose response services such as the National Overdose Response Service (NORS) and Brave, work by engaging with third party virtual supervisors who would monitor the client in their current location, including acute care sites. Some services, such as Lifeguard, are automated with no interaction with PWUD. Due to these unique functional attributes, there were concerns that some ODTs would lead to less face-to-face time with onsite addictions and harm reduction support, limiting a potential therapeutic relationship that would form if there was a consistent engagement between these services and the admitted client.

Feelings were mixed regarding the ability of some ODTs to build rapport and a therapeutic relationship with PWUD, which was seen as a key aspect of managing PWUD. Some interviewees did see this as an opportunity for PWUD, who may not want to engage with onsite addiction staff, to engage with other external resources, such as the peer workers on the NORS hotline. However, many individuals felt as though in-hospital physical SCS would be a better option for PWUD if they were available. Physical SCSs provide additional opportunities for healthcare providers in acute care settings to engage meaningfully with individuals and develop ongoing rapport. This connection could be disrupted by third-party operators such as NORS or Brave. *“I mean whenever you don’t have that face-to-face contact you’re losing an opportunity to engage the person and build those relationships. P12 (Urban Registered Nurse).*

#### Subtheme 3: Stigma reduction

Similarly, interviewees believed that ODTs would allow for stigma reduction for PWUD, demonstrating to them that they are “worthy and important”. *Anytime that we can support people to use substances safely, we do a little bit of work to destigmatize that, and empower people to know that they're worth—they’re worthy and important, and that we want them to stay alive. So, I think that having a virtual service can work towards that goal of destigmatizing substance use, which is what I'd love to see… I think there is a message behind it that states, “we want to keep you safe, we want to keep you healthy, we want to look after you, this is important.” P15 (Urban Harm Reduction Nurse).*

The mobility of ODTs allows flexible implementation in acute care settings and services localized to patient’s rooms. With overdose motion detectors and buttons, they would need to be installed within set locations in which individuals who are assumed to use substances could be placed. One interviewee identified this method of harm reduction as inadvertently stigmatizing by segregating them. This would apply not only to ODTs but also to physical SCSs. *I just think it does inadvertently say, “yeah, we care about you, but if you decide you want to use drugs, please go do it over here with these people, and we’re going to just take care of this section.” And I think when we do that, it is stigmatizing. P15 (Urban Harm Reduction Nurse).*

Taken together, these quotes illustrate that while participants believe ODTs might represent a safe and acceptable harm reduction strategy in healthcare settings, they may be limited in their ability to build rapport and reduce stigma.

### *Section 2: Impacts on healthcare workers*

#### Subtheme 1: Impact of ODTs on healthcare worker burden

*Regarding* how these services would impact healthcare workers, participants believed that these services would likely decrease the financial and human resource strain on participating facilities by freeing up nursing time that otherwise would have been focused on checking in on patients. Indeed, patients at risk of an overdose in their rooms often took nurses away from their other nursing duties and limited their ability to manage multiple patients simultaneously as they were focused on ensuring the wellness of a high-risk patient. *“I think it would help, especially if it came down to like our staffing, right? The emergency room staff are so burned out, so stressed out enough as it is, if there’s a way to implement this sort of service so that would free up staff to not have to worry about, I could see some benefit in that.” P13 (Urban peer support worker).*

#### Subtheme 2: Stigma and buy-in from health care providers

The largest identified barrier to implementing this type of service in acute care settings, and in turn, ensuring it was advertised to patients was buy-in from healthcare providers and leadership. Participants recognized that stigma against PWUD continues to be pervasive within the healthcare system and many healthcare providers continue to feel as though harm reduction initiatives are enabling drug use.

*“I think the biggest barrier would be staff attitude and changing the kind of conversation about people feeling territorial, and oh why would they need that when they're in hospital? Or why are we even supporting substance use and those kind of attitudes.” P5 (Rural Registered Nurse).*

“ A lot of staff are weary to promote something like that because they feel like that’s enabling” P6 (Urban Registered Nurse)

### Section 3: System-level challenges

#### Subtheme 1: Coordination of emergency response

In regard to the feasibility of these services, participants called for additional opportunities to support people who use substances in care such access to opioid agonist treatments, and other harm reduction strategies including access to naloxone kits, and sterile supplies. There were concerns raised about responses in case of emergency or drug poisoning events while using ODTs, and how the response would be coordinated, including who would be conducting the response, whether it be nursing staff, or specialty code teams within the hospital. *Again I think it’s, the biggest reason I can think of is the logistics of the emergency response when it’s needed, how does that work? P3 (Urban Nurse Educator).*

#### Subtheme 2: Policy and legal issues

Many participants “*Would say policy is the biggest (barrier).” P2 (Urban primary care physician).*

Health authorities were identified as being apprehensive about implementing these services in case adverse events are not appropriately attended to or responded to. Indeed, there continue to be voiced concerns about “*promoting drug use and enabling” P12 (Registered Nurse)* and “*who is liable for a patient who is using” P11(P11 Resident Physician)* but in general, participants were still supportive of the main aim of promoting safety in substance use. Similarly, organizations responsible for ensuring the health and safety of staff members may also not be supportive of these services.

*“I think you definitely would have to have a whole overhaul on your policy within a hospital and it would have to have the buy in from like the upper echelons, but also the nursing too. I think even with our supervised injection site opening here at the hospital there was a lot of concern about what their liability was and things like that.” P12 (Urban Registered Nurse).*

#### Subtheme 3: A holistic approach to harm reduction in acute care settings

Lastly, a consistent theme throughout the interviews was that participants identified that ODTs should be implemented as one aspect of a comprehensive harm reduction support system within healthcare settings. Participants recognized a holistic approach is required, including in-person peer support services, sterile supplies, naloxone kits, and options for physical SCS. A combination of services to suite a variety of needs patients may have was felt to be the best option for engaging people who use substances in healthcare settings. “*It needs to be seen as one aspect of the whole plan and not the plan. P14 (Urban health research project lead). Many participants also suggested these services be recommended to patients on discharge planning, potentially in conjunction with the distribution of naloxone (P12 Registered Nurse) or as part of information for individuals to read over an explanation of various services and their usage (P11 Resident Physician).*

*“I think that we, as an organization, can certainly maybe on discharge, recommend these services to folks.” P15 (Regional Nursing Lead).*

Overall, participants expressed mixed opinions about implementing ODTs in acute care settings from a systems-based level. They highlight that there are limitations and barriers to implementing ODT-based services, as well as the importance of a comprehensive model of integrating harm reduction into these settings. Such an integrated and comprehensive approach is core to supporting the healthcare of PWUD accessing healthcare facilities and decreasing stigmatizing attitudes held by healthcare professionals and decision-makers. Furthermore, participants expressed positive attitudes towards ODTs and their inclusion in comprehensive discharge planning.

## Discussion

Based on our evaluation of the perspectives of 16 healthcare professionals, participants held mixed beliefs on implementing ODTs in acute care settings due to various barriers. However, the participants believed that information about these interventions should be provided to individuals with access to technology upon discharge from acute care settings. While participants identified benefits, including improvements in safety for PWUD if using in the hospital alone, there were concerns these services would not be able to build rapport between patients and providers and that its implementation and offering to clients would be impacted by stigma at both provider and administrative levels. Additionally, key barriers to integrating ODTs include access to the technology required by these services, leadership buy-in, liability, and institutional policy.

First and foremost, healthcare workers highlighted the potential for improved safety and well-being for individuals using substances alone in acute care settings. As shown in the literature, ODTs are a tool to reduce overdose deaths and improve community safety [22] and, therefore would provide an additional safety measure for individuals in hospitals, especially when substance use is not disclosed to healthcare professionals. Previous studies on the implementation of passive overdose sensors in washrooms posit that they would provide a more dignified solution to current practices of staff knocking on the door periodically when PWUDs use the bathroom [29]; these sensors are currently being implemented in one hospital in British Columbia [30]. The authors noted during the triangulation process that healthcare providers often do frequent checks on clients suspected of using substances in their rooms to ensure they do not overdose. As a result, efficacious ODTs in healthcare settings may help reduce staff's burden to monitor these spaces continuously.

Some participants discuss both the opportunity and challenges of harm reduction services to build rapport with individuals using substances in the hospital. It has been previously hypothesized that the implementation of passive surveillance technologies would increase awareness of risks associated with substance use and promote community engagement [29]. Some of the aforementioned services, like NORS, the Never Use Alone hotline, and the Brave app [20, 21, 31], further promote connection to the community as the operators are PWUD or have lived experience. This could be especially important in communities that may not have harm reduction supports or peer-led services, allowing patients to connect with services that are not necessarily provided within their communities. Additionally, provided patients have access to technology, they can continue to use the service after discharge from the hospital, maintaining relationships developed with operators of these hotline services. In recent years, peer support workers have been implemented across acute care and have been studied to improve patient engagement and assist with appropriate knowledge translation strategies [32–34] but remain limited in their implementation, particularly in resource-limited settings such as rural hospitals. The delivery and assessment of harm reduction needs for PWUD allow for more significant discussion of substance use goals and have the potential to reduce stigma on behalf of healthcare workers; however, if these systems are overly automated, they may reduce these opportunities and possibly reduce benefits [9]. Future research should evaluate the effectiveness of virtual services in achieving these same outcomes with virtual peer-based support across various resource-limited acute and primary care settings.

One significant barrier highlighted by study participants would be the coordination of emergency responses within acute care settings. Previous studies have described the implementation of bathroom sensors [17] in these spaces, however, these are not seen with other types of ODTs. Careful planning for integrating technologies should allow for appropriate connections between responding healthcare staff. Indeed, due to jurisdictional boundaries, paramedics and other emergency response personnel cannot respond to emergencies within acute care centers. By providing direct lines of communication with hospital staff through nursing desks or other in-hospital rapid response teams, ODTs may facilitate more rapid intervention. Currently, nurses and other clinicians already respond regularly to drug poisoning events in hospitals. Implementing ODTs may help decrease some of the human resource and emotional burdens shouldered by healthcare staff [35].

Liability around these services was also highlighted. Concerns from hospital administration on condoning substance use on-premises were voiced, as well as concerns that if this technology failed, there could be repercussions on hospital staff and administration from a legal standpoint. These are common themes within the harm reduction space, particularly in acute care settings [9, 29]. This barrier would be enough to discourage some health authorities and hospital administrations from implementing these services.

While consensus was mixed about the utility of virtual harm reduction services within acute care settings, most participants highlighted the importance of including these services as part of discharge planning, harm reduction education, and provision of take-home naloxone kits. Indeed, there is a significant increase in the risk of overdose upon discharge from the hospital, and it is therefore integral that individuals are directed to appropriate support [36]. Current discharge planning across Canada often does not include harm reduction strategies beyond providing a naloxone kit.

These services may help promote the safety of PWUD in acute care settings and may help to change the pervasive stigma these individuals face in accessing care. As highlighted by participants, these ODTs should be used as an adjunct option to other currently available services such as peer support workers [34], in-hospital supervised consumption services [14, 15], and access to ongoing community supports, including opioid agonist and addiction treatment clinics [37]**.**

Additionally, there could be situations where technology-based overdose prevention services could be helpful. For instance, if individuals are isolated within the hospital and cannot make it to a physical onsite SCS, ODTs could be an option. Additionally, if PWUD do not self-disclose or do not want to disclose their substance use, ODTs, especially sensors, could be used for clients who are deemed high risk of a drug poisoning event but may increase stigma towards this population [29]. In addition, sensors may be a reasonable option in more public spaces in acute care sites such as public washrooms or washrooms in spaces like emergency departments where numerous individuals use space and high frequency of poisoning events are recorded.

It was noted that while ODTs could reduce stigma and create border acceptance of harm reduction in hospital settings, it was also felt by some participants that referring people to different services could make individuals feel as though they were being passed along and not supported by hospital staff. Thus, a combination of solutions wherein they could feel supported by hospital staff but also have the option to reach out to outside support is likely the best option. These services would be ideal first steps for acute care centers to support PWUD; however, ODTs may be more feasible due to the decreased costs associated with promoting established services.

Additional research should examine the impact of ODTs in outcomes once implemented including any changes to the patient-described quality of care by clients and staff after incorporating these technologies. Furthermore, while these services have demonstrated their acceptability in qualitative research in PWUD populations [38, 39], further examination of the acceptability of these services in acute care settings should be undertaken. Additional research on virtual peer interventions may have additional benefits in line with those previously described by studies of peer navigators in healthcare settings, such as patient advocacy and reductions in barriers to care [40].

### Limitations

Due to the broad geographic scope of our study, both perspectives of healthcare workers on illicit substance use, the amount of harm reduction programming implemented already in their setting, and policies regarding substance use varied significantly, which may contribute to the diverse sentiments on the implementation of these services in acute care settings. Similarly, to our knowledge, there are no Canadian statistics on overdose within healthcare settings in Canada, which may limit the applicability of our findings. Reporting of statistics on fatal overdoses within acute care settings s would be beneficial to inform decision-makers regarding the potential need for additional ODTs or on-site harm reduction services.

Our analysis was limited to the perspectives of healthcare workers on implementing overdose detection technologies in acute care settings. Additional research should look to evaluate the perspectives of those most impacted by its implementation, namely PWUD in acute care settings. Furthermore, studies should also evaluate the outcomes of service implementation and resultant changes in self-initiating discharge and health outcomes for this target population. The sampling strategy used within our analysis, namely convenience and snowball sampling, may have reduced the diversity of opinions in the sample. However, attempts were made to recruit geographically and demographically diverse participants.

## Conclusion

Overdose detection technologies were perceived as an acceptable intervention to implement within acute care settings as part of a larger harm reduction framework. Potential benefits of service implementation include increased patient safety and reductions in stigma. Participants provided mixed perspectives on the efficacy of these services in building rapport with PWUD. Lastly, many barriers would likely exist in implementing the various programs noted, including staff attitudes and legal issues.

### Supplementary Information

**Additional file 1.** Additional quotes from the qualitative interviews.

## Data Availability

The data that support the findings of this study are available on request from the corresponding author, M.G. The data are not publicly available due to the sensitivity of substance use and interview transcripts containing information that could compromise the privacy of research participants.

## Abbreviations

ODTs: Virtual overdose monitoring services
PWUD: People who use drugs
NORS: National overdose response service
DORS: Digital overdose response system
SCS: Supervised consumption sites
